# Di-μ-methoxido-μ-oxido-bis­[triphenyl­antimony(V)] methanol disolvate

**DOI:** 10.1107/S1600536809052192

**Published:** 2009-12-09

**Authors:** Richard Betz, Sebastian Junggeburth, Peter Klüfers, Peter Mayer

**Affiliations:** a Ludwig-Maximilians Universität, Department Chemie und Biochemie, Butenandtstrasse 5–13 (Haus D), 81377 München, Germany

## Abstract

The title compound, [Sb_2_(C_6_H_5_)_6_(CH_3_O)_2_O]·2CH_3_OH, is the methanol disolvate of a dinuclear triphenyl­anti­mony derivative. The mol­ecule shows *C*_s_ symmetry. The Sb—O—Sb angles cover a range from 89.65 (10)° to 102.08 (13)°. In the crystal structure, two O—H⋯O hydrogen bonds are present.

## Related literature

For related structures, see: Bordner *et al.* (1986 ▶). For graph-set analysis, see: Bernstein *et al.* (1995 ▶); Etter *et al.* (1990 ▶). For the synthesis of triphenyl­stibane oxide, see: Goodgame & Cotton (1960 ▶).
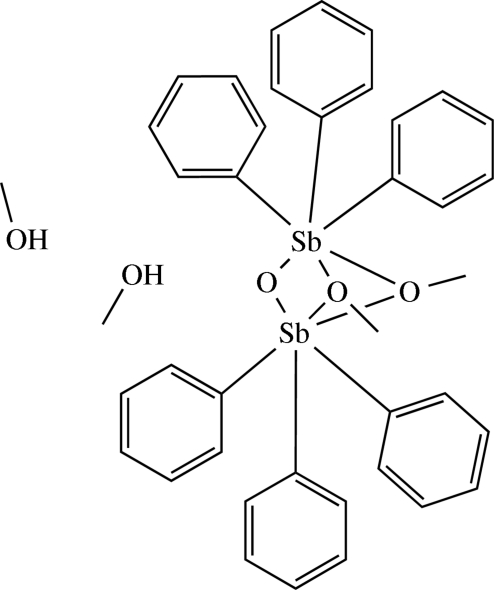


## Experimental

### 

#### Crystal data

[Sb_2_(C_6_H_5_)_6_(CH_3_O)_2_O]·2CH_4_O
*M* *_r_* = 848.25
Orthorhombic, 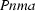

*a* = 10.7666 (3) Å
*b* = 20.9205 (5) Å
*c* = 16.5619 (5) Å
*V* = 3730.45 (18) Å^3^
*Z* = 4
Mo *K*α radiation
μ = 1.49 mm^−1^
*T* = 200 K
0.21 × 0.13 × 0.11 mm

#### Data collection

Oxford Xcalibur KappaCCD diffractometer
Absorption correction: multi-scan (*CrysAlis RED*; Oxford Diffraction, 2005 ▶) *T*_min_ = 0.923, *T*_max_ = 1.000
15055 measured reflections
3877 independent reflections
2632 reflections with *I* > 2σ(*I*)
*R*_int_ = 0.035

#### Refinement

*R*[*F* ^2^ > 2σ(*F* ^2^)] = 0.025
*wR*(*F* ^2^) = 0.069
*S* = 1.07
3877 reflections
250 parameters
35 restraints
H atoms treated by a mixture of independent and constrained refinement
Δρ_max_ = 1.16 e Å^−3^
Δρ_min_ = −0.91 e Å^−3^

### 

Data collection: *CrysAlis CCD* (Oxford Diffraction, 2005 ▶); cell refinement: *CrysAlis RED* (Oxford Diffraction, 2005 ▶); data reduction: *CrysAlis RED*; program(s) used to solve structure: *SHELXS97* (Sheldrick, 2008 ▶); program(s) used to refine structure: *SHELXL97* (Sheldrick, 2008 ▶); molecular graphics: *ORTEP*-III (Farrugia, 1997 ▶); software used to prepare material for publication: *SHELXL97*.

## Supplementary Material

Crystal structure: contains datablocks I, global. DOI: 10.1107/S1600536809052192/bq2171sup1.cif

Structure factors: contains datablocks I. DOI: 10.1107/S1600536809052192/bq2171Isup2.hkl

Additional supplementary materials:  crystallographic information; 3D view; checkCIF report

## Figures and Tables

**Table 1:** Hydrogen-bond geometry (Å, °)

*D*—H⋯*A*	*D*—H	H⋯*A*	*D*⋯*A*	*D*—H⋯*A*
O90—H90*C*⋯O1	0.83 (2)	1.90 (3)	2.718 (5)	168 (7)
O91—H91*C*⋯O90	0.84 (2)	1.81 (2)	2.642 (7)	180 (15)

**Fig. 1.**
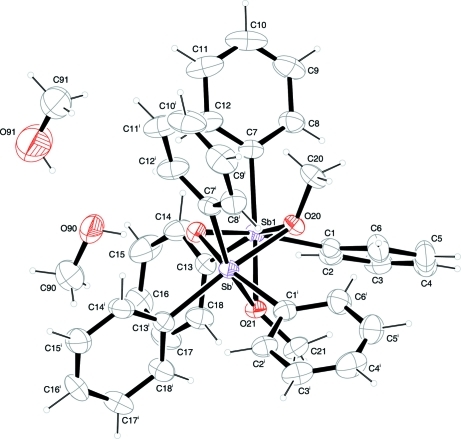
The molecular structure of the title compound, with atom labels and anisotropic displacement ellipsoids (drawn at 50% probability level) for non-H atoms. Symmetry code: i x, -y + 1/2, z.

**Fig. 2.**
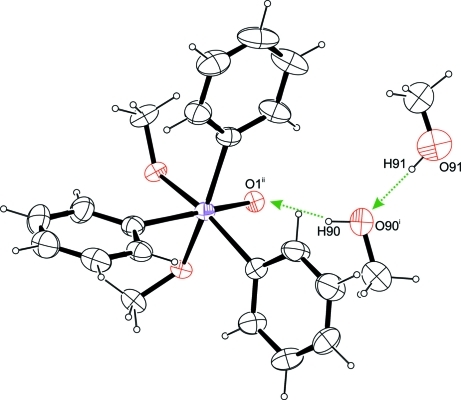
Hydrogen bonds in the crystal structure of the title compound, viewed along [0 1 0]. Symmetry codes: i x + 1/2, y, -z + 1/2; ii x - 1/2, y, -z + 1/2.

## supplementary crystallographic information

### Comment

In a program focused on the synthesis of carbohydrate-derived chelates of members of the *p*-block of the periodic table of elements, efforts were made to synthesize a diol compound derived from Sb(V). Unintendedly, a dinuclear triphenylantimony derivative was isolated.

In the *C*_s_ symmetric molecule, two antimony atoms bearing three phenyl moieties each are connected by two methoxido ligands and one oxido ligand (Fig. 1). The Sb–O–Sb angles cover a range from 90° to 102° with the largest angle on the oxido bridge.

In the crystal structure, two methanol molecules are present which form finite hydrogen bonds (Fig. 2). On the unitary level of graph-set analysis (Etter *et al.*, 1990; Bernstein *et al.*, 1995) the descriptor of this pattern is DD.

### Experimental

The compound was unintendedly prepared upon the attempted condensation of triphenylstibane oxide with a polyol in an aprotic solvent. 1 eq. of triphenylstibane oxide (prepared according to Goodgame & Cotton, 1960) was stirred with 1 eq. of xylitol in dioxane until a clear solution was obtained. After removal of the solvent and subsequent recrystallization from methanol crystals were obtained.

### Refinement

H atoms bonded to aromatic C atoms were placed in calculated positions (C—H 0.95 Å) and were included in the refinement in the riding model approximation, with *U*(H) set to 1.2*U*_eq_(C). H atoms bonded to methyl groups and hydroxyl groups were refined freely with fixed distances using *DFIX* instructions (C—H 0.96 (2) Å; O—H 0.83 (2) Å). For the refinement their *U*(H) was set to 1.5*U*_eq_(C) and 1.5*U*_eq_(O), respectively. The C and O atoms of the free methanol molecules were refined so that their *U_ij_* components approximate to isotropic behaviour.

### Crystal data

**Table:**

[Sb_2_(C_6_H_5_)_6_(CH_3_O)_2_O]·2CH_4_O	*F*(000) = 1704
*M**_r_* = 848.25	*D*_x_ = 1.510 Mg m^−^^3^
Orthorhombic, *P**n**m**a*	Mo *K*α radiation, λ = 0.71073 Å
Hall symbol: -P 2ac 2n	Cell parameters from 6848 reflections
*a* = 10.7666 (3) Å	θ = 3.8–26.3°
*b* = 20.9205 (5) Å	µ = 1.49 mm^−^^1^
*c* = 16.5619 (5) Å	*T* = 200 K
*V* = 3730.45 (18) Å^3^	Rod, colourless
*Z* = 4	0.21 × 0.13 × 0.11 mm

### Data collection

**Table:**

Oxford Xcalibur KappaCCD diffractometer	3877 independent reflections
Radiation source: fine-focus sealed tube	2632 reflections with *I* > 2σ(*I*)
graphite	*R*_int_ = 0.035
ω–scans	θ_max_ = 26.3°, θ_min_ = 3.8°
Absorption correction: multi-scan (*CrysAlis RED*; Oxford Diffraction, 2005)	*h* = −13→10
*T*_min_ = 0.923, *T*_max_ = 1.000	*k* = −25→26
15055 measured reflections	*l* = −20→20

### Refinement

**Table:**

Refinement on *F*^2^	Primary atom site location: structure-invariant direct methods
Least-squares matrix: full	Secondary atom site location: difference Fourier map
*R*[*F*^2^ > 2σ(*F*^2^)] = 0.025	Hydrogen site location: inferred from neighbouring sites
*wR*(*F*^2^) = 0.069	H atoms treated by a mixture of independent and constrained refinement
*S* = 1.07	*w* = 1/[σ^2^(*F*_o_^2^) + (0.0343*P*)^2^] where *P* = (*F*_o_^2^ + 2*F*_c_^2^)/3
3877 reflections	(Δ/σ)_max_ = 0.001
250 parameters	Δρ_max_ = 1.16 e Å^−^^3^
35 restraints	Δρ_min_ = −0.91 e Å^−^^3^

### Special details

**Table:**

Experimental. Absorption correction Empirical absorption correction using spherical harmonics, implemented in SCALE3 ABSPACK scaling algorithm.

### Fractional atomic coordinates and isotropic or equivalent isotropic displacement parameters (Å^2^)

**Table:**

	*x*	*y*	*z*	*U*_iso_*/*U*_eq_	
C1	0.3088 (3)	0.12147 (15)	0.46497 (18)	0.0318 (7)	
C2	0.3241 (3)	0.06249 (15)	0.42762 (19)	0.0351 (8)	
H2	0.4048	0.0494	0.4112	0.042*	
C3	0.2232 (3)	0.02236 (17)	0.4139 (2)	0.0445 (9)	
H3	0.2349	−0.0178	0.3883	0.053*	
C4	0.1068 (4)	0.04132 (18)	0.4375 (2)	0.0535 (11)	
H4	0.0374	0.0143	0.4281	0.064*	
C5	0.0904 (4)	0.0994 (2)	0.4748 (2)	0.0565 (11)	
H5	0.0094	0.1121	0.4910	0.068*	
C6	0.1907 (3)	0.13995 (18)	0.4893 (2)	0.0448 (9)	
H6	0.1784	0.1798	0.5156	0.054*	
C7	0.5293 (3)	0.13287 (13)	0.59936 (18)	0.0302 (7)	
C8	0.4591 (3)	0.08530 (16)	0.6357 (2)	0.0420 (9)	
H8	0.3868	0.0696	0.6096	0.050*	
C9	0.4943 (4)	0.06062 (19)	0.7100 (2)	0.0543 (11)	
H9	0.4457	0.0281	0.7346	0.065*	
C10	0.5977 (5)	0.08263 (18)	0.7479 (2)	0.0640 (13)	
H10	0.6195	0.0664	0.7995	0.077*	
C11	0.6706 (4)	0.12803 (17)	0.7119 (2)	0.0580 (12)	
H11	0.7445	0.1421	0.7377	0.070*	
C12	0.6364 (4)	0.15360 (16)	0.6376 (2)	0.0455 (10)	
H12	0.6868	0.1854	0.6130	0.055*	
C13	0.5940 (3)	0.13122 (14)	0.40116 (18)	0.0289 (7)	
C14	0.6844 (3)	0.08906 (17)	0.4273 (2)	0.0450 (9)	
H14	0.6913	0.0797	0.4832	0.054*	
C15	0.7646 (4)	0.06037 (19)	0.3733 (2)	0.0597 (11)	
H15	0.8258	0.0312	0.3919	0.072*	
C16	0.7554 (3)	0.07419 (18)	0.2925 (2)	0.0521 (10)	
H16	0.8121	0.0555	0.2555	0.063*	
C17	0.6651 (4)	0.11463 (16)	0.2649 (2)	0.0464 (9)	
H17	0.6579	0.1232	0.2088	0.056*	
C18	0.5844 (4)	0.14299 (15)	0.3193 (2)	0.0402 (8)	
H18	0.5214	0.1709	0.3000	0.048*	
O1	0.5888 (3)	0.2500	0.50194 (15)	0.0258 (7)	
O20	0.3612 (3)	0.2500	0.54656 (17)	0.0277 (7)	
O21	0.4204 (3)	0.2500	0.40014 (16)	0.0264 (6)	
Sb1	0.47467 (2)	0.175954 (8)	0.487384 (12)	0.02407 (8)	
C20	0.3345 (5)	0.2500	0.6305 (3)	0.0400 (13)	
H20A	0.407 (5)	0.2500	0.660 (3)	0.060*	
H20B	0.283 (3)	0.2876 (16)	0.645 (2)	0.060*	
C21	0.3109 (5)	0.2500	0.3525 (3)	0.0397 (12)	
H21A	0.237 (3)	0.2500	0.385 (3)	0.060*	
H21B	0.299 (3)	0.2858 (12)	0.3186 (18)	0.060*	
C90	0.8713 (6)	0.2500	0.3896 (4)	0.0681 (18)	
O90	0.8368 (3)	0.2500	0.4711 (3)	0.0744 (13)	
H90A	0.960 (2)	0.2500	0.385 (5)	0.112*	
H90B	0.839 (4)	0.2871 (16)	0.363 (3)	0.112*	
H90C	0.7602 (19)	0.2500	0.473 (4)	0.112*	
C91	0.9684 (6)	0.2500	0.6588 (5)	0.100 (3)	
O91	1.0052 (5)	0.2500	0.5871 (4)	0.176 (4)	
H91A	1.038 (3)	0.2500	0.6941 (16)	0.264*	
H91B	0.916 (3)	0.2124 (5)	0.665 (2)	0.264*	
H91C	0.952 (10)	0.2500	0.550 (6)	0.264*	

### Atomic displacement parameters (Å^2^)

**Table:**

	*U*^11^	*U*^22^	*U*^33^	*U*^12^	*U*^13^	*U*^23^
C1	0.0364 (19)	0.0323 (17)	0.0268 (18)	−0.0047 (16)	−0.0006 (14)	0.0040 (13)
C2	0.044 (2)	0.0330 (17)	0.0287 (18)	−0.0055 (17)	−0.0045 (16)	0.0053 (14)
C3	0.061 (2)	0.0344 (19)	0.038 (2)	−0.014 (2)	−0.0098 (18)	0.0050 (15)
C4	0.052 (3)	0.060 (3)	0.048 (2)	−0.031 (2)	−0.004 (2)	0.0075 (19)
C5	0.035 (2)	0.075 (3)	0.059 (3)	−0.015 (2)	0.007 (2)	−0.003 (2)
C6	0.039 (2)	0.051 (2)	0.045 (2)	−0.0101 (19)	0.0073 (18)	−0.0066 (17)
C7	0.0411 (19)	0.0259 (16)	0.0236 (16)	0.0067 (16)	0.0000 (16)	−0.0009 (12)
C8	0.047 (2)	0.0409 (19)	0.038 (2)	0.0017 (18)	−0.0001 (18)	0.0110 (16)
C9	0.075 (3)	0.044 (2)	0.043 (2)	0.006 (2)	0.009 (2)	0.0178 (18)
C10	0.114 (4)	0.047 (2)	0.030 (2)	0.023 (3)	−0.009 (2)	0.0055 (19)
C11	0.088 (3)	0.042 (2)	0.043 (2)	0.005 (2)	−0.029 (2)	−0.0004 (18)
C12	0.068 (3)	0.0330 (19)	0.036 (2)	−0.0012 (19)	−0.0146 (19)	0.0029 (15)
C13	0.0324 (18)	0.0252 (16)	0.0291 (17)	−0.0018 (14)	0.0025 (15)	−0.0015 (13)
C14	0.054 (2)	0.053 (2)	0.0282 (19)	0.016 (2)	−0.0059 (18)	−0.0065 (16)
C15	0.058 (3)	0.071 (3)	0.050 (3)	0.033 (2)	−0.009 (2)	−0.016 (2)
C16	0.050 (2)	0.059 (3)	0.047 (2)	0.013 (2)	0.0143 (19)	−0.0154 (19)
C17	0.067 (3)	0.040 (2)	0.032 (2)	−0.001 (2)	0.0106 (19)	−0.0052 (16)
C18	0.053 (2)	0.0347 (19)	0.033 (2)	0.0088 (18)	0.0024 (18)	0.0016 (15)
O1	0.0259 (16)	0.0229 (14)	0.0284 (17)	0.000	−0.0009 (13)	0.000
O20	0.0316 (17)	0.0268 (15)	0.0246 (16)	0.000	0.0054 (14)	0.000
O21	0.0302 (16)	0.0277 (16)	0.0213 (15)	0.000	−0.0026 (13)	0.000
Sb1	0.02763 (12)	0.02247 (11)	0.02209 (12)	−0.00063 (10)	0.00017 (9)	0.00018 (8)
C20	0.051 (3)	0.037 (3)	0.032 (3)	0.000	0.016 (2)	0.000
C21	0.040 (3)	0.042 (3)	0.037 (3)	0.000	−0.014 (2)	0.000
C90	0.058 (4)	0.089 (5)	0.057 (4)	0.000	0.012 (4)	0.000
O90	0.034 (2)	0.140 (4)	0.049 (3)	0.000	−0.001 (2)	0.000
C91	0.062 (5)	0.182 (9)	0.056 (5)	0.000	0.007 (4)	0.000
O91	0.077 (4)	0.385 (12)	0.064 (4)	0.000	0.003 (3)	0.000

### Geometric parameters (Å, °)

**Table:**

C1—C6	1.388 (5)	C15—C16	1.372 (5)
C1—C2	1.390 (4)	C15—H15	0.9500
C1—Sb1	2.151 (3)	C16—C17	1.367 (5)
C2—C3	1.392 (4)	C16—H16	0.9500
C2—H2	0.9500	C17—C18	1.386 (4)
C3—C4	1.372 (5)	C17—H17	0.9500
C3—H3	0.9500	C18—H18	0.9500
C4—C5	1.375 (5)	O1—Sb1	1.9921 (18)
C4—H4	0.9500	O1—Sb1^i^	1.9922 (18)
C5—C6	1.394 (5)	O20—C20	1.420 (5)
C5—H5	0.9500	O20—Sb1	2.203 (2)
C6—H6	0.9500	O20—Sb1^i^	2.203 (2)
C7—C12	1.386 (4)	O21—C21	1.419 (5)
C7—C8	1.387 (4)	O21—Sb1^i^	2.1975 (19)
C7—Sb1	2.144 (3)	O21—Sb1	2.1975 (19)
C8—C9	1.388 (5)	Sb1—Sb1^i^	3.0981 (4)
C8—H8	0.9500	C20—H20A	0.92 (6)
C9—C10	1.358 (5)	C20—H20B	0.99 (3)
C9—H9	0.9500	C21—H21A	0.956 (19)
C10—C11	1.368 (5)	C21—H21B	0.945 (17)
C10—H10	0.9500	C90—O90	1.400 (8)
C11—C12	1.391 (5)	C90—H90A	0.96 (2)
C11—H11	0.9500	C90—H90B	0.959 (19)
C12—H12	0.9500	O90—H90C	0.83 (2)
C13—C18	1.382 (4)	C91—O91	1.252 (9)
C13—C14	1.383 (4)	C91—H91A	0.95 (2)
C13—Sb1	2.137 (3)	C91—H91B	0.973 (18)
C14—C15	1.381 (5)	O91—H91C	0.84 (2)
C14—H14	0.9500		
			
C6—C1—C2	119.0 (3)	C16—C17—C18	119.6 (3)
C6—C1—Sb1	124.2 (2)	C16—C17—H17	120.2
C2—C1—Sb1	116.6 (2)	C18—C17—H17	120.2
C1—C2—C3	121.0 (3)	C13—C18—C17	121.0 (3)
C1—C2—H2	119.5	C13—C18—H18	119.5
C3—C2—H2	119.5	C17—C18—H18	119.5
C4—C3—C2	119.5 (3)	Sb1—O1—Sb1^i^	102.08 (13)
C4—C3—H3	120.3	C20—O20—Sb1	123.25 (17)
C2—C3—H3	120.3	C20—O20—Sb1^i^	123.25 (17)
C3—C4—C5	120.1 (4)	Sb1—O20—Sb1^i^	89.38 (10)
C3—C4—H4	120.0	C21—O21—Sb1^i^	125.92 (15)
C5—C4—H4	120.0	C21—O21—Sb1	125.92 (15)
C4—C5—C6	121.1 (4)	Sb1^i^—O21—Sb1	89.65 (10)
C4—C5—H5	119.5	O1—Sb1—C13	92.90 (11)
C6—C5—H5	119.5	O1—Sb1—C7	93.03 (11)
C1—C6—C5	119.3 (3)	C13—Sb1—C7	103.24 (11)
C1—C6—H6	120.3	O1—Sb1—C1	160.95 (11)
C5—C6—H6	120.3	C13—Sb1—C1	98.73 (12)
C12—C7—C8	118.7 (3)	C7—Sb1—C1	98.88 (12)
C12—C7—Sb1	119.5 (2)	O1—Sb1—O21	72.27 (9)
C8—C7—Sb1	121.8 (2)	C13—Sb1—O21	91.67 (10)
C7—C8—C9	120.2 (4)	C7—Sb1—O21	159.72 (9)
C7—C8—H8	119.9	C1—Sb1—O21	92.25 (10)
C9—C8—H8	119.9	O1—Sb1—O20	75.01 (9)
C10—C9—C8	120.4 (4)	C13—Sb1—O20	159.85 (10)
C10—C9—H9	119.8	C7—Sb1—O20	93.61 (10)
C8—C9—H9	119.8	C1—Sb1—O20	89.38 (10)
C9—C10—C11	120.3 (4)	O21—Sb1—O20	69.47 (9)
C9—C10—H10	119.8	O1—Sb1—Sb1^i^	38.96 (6)
C11—C10—H10	119.8	C13—Sb1—Sb1^i^	115.98 (8)
C10—C11—C12	120.0 (4)	C7—Sb1—Sb1^i^	114.86 (8)
C10—C11—H11	120.0	C1—Sb1—Sb1^i^	122.00 (9)
C12—C11—H11	120.0	O21—Sb1—Sb1^i^	45.18 (5)
C7—C12—C11	120.3 (4)	O20—Sb1—Sb1^i^	45.31 (5)
C7—C12—H12	119.9	O20—C20—H20A	110 (3)
C11—C12—H12	119.9	O20—C20—H20B	110 (2)
C18—C13—C14	118.2 (3)	H20A—C20—H20B	111 (3)
C18—C13—Sb1	122.2 (2)	O21—C21—H21A	112 (3)
C14—C13—Sb1	119.6 (2)	O21—C21—H21B	116 (2)
C15—C14—C13	121.0 (3)	H21A—C21—H21B	103 (3)
C15—C14—H14	119.5	O90—C90—H90A	110 (5)
C13—C14—H14	119.5	O90—C90—H90B	111 (3)
C16—C15—C14	119.6 (4)	H90A—C90—H90B	109 (4)
C16—C15—H15	120.2	C90—O90—H90C	108 (5)
C14—C15—H15	120.2	O91—C91—H91A	109 (2)
C17—C16—C15	120.5 (3)	O91—C91—H91B	106 (2)
C17—C16—H16	119.8	H91A—C91—H91B	114 (3)
C15—C16—H16	119.8	C91—O91—H91C	118 (10)
			
C6—C1—C2—C3	0.6 (5)	C8—C7—Sb1—C13	−102.7 (3)
Sb1—C1—C2—C3	177.0 (2)	C12—C7—Sb1—C1	179.6 (3)
C1—C2—C3—C4	0.0 (5)	C8—C7—Sb1—C1	−1.5 (3)
C2—C3—C4—C5	−0.3 (5)	C12—C7—Sb1—O21	−57.9 (5)
C3—C4—C5—C6	0.0 (6)	C8—C7—Sb1—O21	121.0 (3)
C2—C1—C6—C5	−0.8 (5)	C12—C7—Sb1—O20	−90.5 (3)
Sb1—C1—C6—C5	−177.0 (3)	C8—C7—Sb1—O20	88.4 (3)
C4—C5—C6—C1	0.6 (6)	C12—C7—Sb1—Sb1^i^	−48.8 (3)
C12—C7—C8—C9	1.9 (5)	C8—C7—Sb1—Sb1^i^	130.0 (2)
Sb1—C7—C8—C9	−177.0 (3)	C6—C1—Sb1—O1	−35.9 (5)
C7—C8—C9—C10	−0.2 (6)	C2—C1—Sb1—O1	147.8 (3)
C8—C9—C10—C11	−2.0 (6)	C6—C1—Sb1—C13	−162.9 (3)
C9—C10—C11—C12	2.4 (6)	C2—C1—Sb1—C13	20.8 (2)
C8—C7—C12—C11	−1.5 (5)	C6—C1—Sb1—C7	92.1 (3)
Sb1—C7—C12—C11	177.4 (3)	C2—C1—Sb1—C7	−84.1 (2)
C10—C11—C12—C7	−0.6 (6)	C6—C1—Sb1—O21	−70.8 (3)
C18—C13—C14—C15	1.3 (5)	C2—C1—Sb1—O21	112.9 (2)
Sb1—C13—C14—C15	−178.7 (3)	C6—C1—Sb1—O20	−1.4 (3)
C13—C14—C15—C16	0.5 (6)	C2—C1—Sb1—O20	−177.7 (2)
C14—C15—C16—C17	−1.9 (6)	C6—C1—Sb1—Sb1^i^	−34.7 (3)
C15—C16—C17—C18	1.5 (6)	C2—C1—Sb1—Sb1^i^	149.0 (2)
C14—C13—C18—C17	−1.7 (5)	C21—O21—Sb1—O1	−169.6 (3)
Sb1—C13—C18—C17	178.2 (3)	Sb1^i^—O21—Sb1—O1	−33.56 (9)
C16—C17—C18—C13	0.3 (5)	C21—O21—Sb1—C13	97.9 (3)
Sb1^i^—O1—Sb1—C13	129.41 (12)	Sb1^i^—O21—Sb1—C13	−126.07 (11)
Sb1^i^—O1—Sb1—C7	−127.15 (12)	C21—O21—Sb1—C7	−124.4 (4)
Sb1^i^—O1—Sb1—C1	1.7 (4)	Sb1^i^—O21—Sb1—C7	11.6 (4)
Sb1^i^—O1—Sb1—O21	38.58 (10)	C21—O21—Sb1—C1	−0.9 (3)
Sb1^i^—O1—Sb1—O20	−34.23 (11)	Sb1^i^—O21—Sb1—C1	135.13 (11)
C18—C13—Sb1—O1	−90.0 (3)	C21—O21—Sb1—O20	−89.4 (3)
C14—C13—Sb1—O1	89.9 (3)	Sb1^i^—O21—Sb1—O20	46.64 (9)
C18—C13—Sb1—C7	176.2 (3)	C21—O21—Sb1—Sb1^i^	−136.1 (3)
C14—C13—Sb1—C7	−3.9 (3)	C20—O20—Sb1—O1	−100.6 (3)
C18—C13—Sb1—C1	74.9 (3)	Sb1^i^—O20—Sb1—O1	29.84 (9)
C14—C13—Sb1—C1	−105.2 (3)	C20—O20—Sb1—C13	−155.3 (3)
C18—C13—Sb1—O21	−17.6 (3)	Sb1^i^—O20—Sb1—C13	−24.9 (4)
C14—C13—Sb1—O21	162.3 (3)	C20—O20—Sb1—C7	−8.4 (3)
C18—C13—Sb1—O20	−37.8 (5)	Sb1^i^—O20—Sb1—C7	122.01 (11)
C14—C13—Sb1—O20	142.1 (3)	C20—O20—Sb1—C1	90.4 (3)
C18—C13—Sb1—Sb1^i^	−57.3 (3)	Sb1^i^—O20—Sb1—C1	−139.13 (11)
C14—C13—Sb1—Sb1^i^	122.6 (2)	C20—O20—Sb1—O21	−176.9 (3)
C12—C7—Sb1—O1	−15.3 (3)	Sb1^i^—O20—Sb1—O21	−46.50 (9)
C8—C7—Sb1—O1	163.6 (3)	C20—O20—Sb1—Sb1^i^	−130.4 (3)
C12—C7—Sb1—C13	78.4 (3)		

Symmetry codes: (i) *x*, −*y*+1/2, *z*.

### Hydrogen-bond geometry (Å, °)

**Table:**

*D*—H···*A*	*D*—H	H···*A*	*D*···*A*	*D*—H···*A*
O90—H90C···O1	0.83 (2)	1.90 (3)	2.718 (5)	168 (7)
O91—H91C···O90	0.84 (2)	1.81 (2)	2.642 (7)	180 (15)
